# Hoof Culture

**Published:** 1892-06

**Authors:** Williamson Bryden


### HOOF CULTURE.

Now that horse breeding has attained such vast proportions, the cultivation of the hoof in domestication has become a study of special importance and interest. In remote times, attention was called to the horse's hoofs, from the fact, that on some kinds of ground, and on highways—in expeditions and in ordinary work—their liability to become worn and foot-sore before the animal was otherwise exhausted, compelled the application of some protection. This has ultimated in the horse-shoes of to-day. A high degree of skill in making them has been attained, which is to be hoped will be still further improved, by a better knowledge of their application as well.

For ages, different districts and countries have been celebrated for their breeds of horses of great speed and endurance—the superiority of these animals being principally due to the form, quality, and endurance of their hoofs; their speed, disposition, and general conformation being also excellent. In studying the principal features of such countries, much will be found to have depended on the climate—whether hot or cold, wet or dry; and on the soil—whether sand, meadow, swamp, hilly and broken ground, or upland; for the surroundings that predominate when the animal is young, determine in a great degree, first, the character of the hoofs peculiar to the locality, and then the quality and form of his limbs peculiar to such hoofs, and the general conformation of his whole body.

In such a country, the animal must have sufficient food, but not in such abundance but that he is compelled to search for it, perhaps long distances. This insures, not only the uniform *tear and wear* of his hoofs, necessary to their robust growth and organization, but it expands his lungs, strenghtens his circulation, and develops his limbs till his speed and endurance are in harmony with a degree of organization the average of which is unequalled. "Function makes the organ," and in suitable surroundings, or under proper management, every essential part of the animal ought to develop symmetrically.

The rearing of young, finely-bred horses, as practiced in domestication at the present time, demands, therefore, the most careful study of the animal, not only in his natural conditions, but under his nomadic surroundings, and his still more recent experiences and methods in domestication. We would ascribe little wisdom to one claiming to be an astronomer who had never seen the starry firmament but with his naked eye, or though ordinary spectacles ; yet, what will be said either of the practitioner, or those who employ him, who regard the hoof merely as a protection to the parts inclosed within it, which nature always provided ; a theory that has long been orthodox, and is still popular in practice, even in our colleges, if one may judge from analyses of their treatment. To study such an organism, within such narrow limits, can never aid us in rising above the unphilosophic ruts in which Veterinarians have so long been laboring, with regard to the influence of the hoofs of solipeds.

The influence of the hoofs dominate every function connected with locomotion, and when imperfect, either from disease or neglect, it determines the character of all acquired diseases peculiar to the horse's locomotive organs. Its *study*, therefore, must be very broad and comprehensive, for it deals with the young animal's wants in domestication, and under restraint, as well as in a state of nature, and in different surroundings. It must show when Art ought to step in, and what should be done to correct irregular growths and derelict conditions of the colt's little hoofs during a winter's confinement ; how to trim them, so that they will grow in right directions, and in such a way that the coffin bone will retain its normal position, and the circulation around the coronet be unembarrassed and free, producing a healthy growth of every part of the "horny box" and its contents, relieving it thereby from much of the liability to induce diseases of the limbs above.

The need of a knowledge of this subject is felt among horse-breeders everywhere. yet our profession neglects to take it up. That heredity and congenital influences are important factors in horse breeding. operating in both desirable and wrong directions, cannot be denied, and consequently, demand careful attention; yet, the surroundings and management from birth to maturity of our young horses seem to me a study of, if possible, the greatest importance, so far as the hoofs are concerned, of any other feature at the present time; on their emerging from winter quarters, the selection of proper pastuage must not be neglected, it is an important matter that ought to receive the strictest attention, for some farms have pasture fields as unfit for colts' feet after months of confinement, as a swamp would be for corn; the little, dried-up hoofs being of all conceivable forms and qualities.

WILLIAMSON BRYDEN, V. S.

